# DaRenCa risk score: A prognostic model for recurrence in clear cell renal cell carcinoma

**DOI:** 10.1002/bco2.70234

**Published:** 2026-06-03

**Authors:** Philip Hedegaard, Rasmus D. Petersson, Hayder Al‐Husseinawi, Emma Heeno, Marina Lunetcas, Sara Tolouee, Rasmine Bak, Ole B. Pedersen, Juan L. Vásquez, Lars Lund, Frederik F. Thomsen

**Affiliations:** ^1^ Department of Urology Copenhagen University Hospital – Zealand University Hospital Roskilde Roskilde Denmark; ^2^ Department of Urology Aalborg University Hospital Aalborg Denmark; ^3^ Department of Urology Odense University Hospital Odense Denmark; ^4^ Department of Radiology Copenhagen University Hospital – Zealand University Hospital Roskilde Roskilde Denmark; ^5^ Department of Urology Copenhagen University Hospital – Herlev‐Gentofte Hospital Gentofte Denmark; ^6^ Department of Urology Aarhus University Hospital Aarhus Denmark; ^7^ Department of Clinical Immunology Copenhagen University Hospital – Zealand University Hospital Køge Køge Denmark; ^8^ Department of Clinical Medicine University of Copenhagen Copenhagen Denmark

**Keywords:** clear cell, machine learning, recurrence, renal cell carcinoma, risk score, surgery

## Abstract

**Objective:**

This study aimed to investigate whether a machine‐learning model improves the assessment of postsurgical recurrence‐free survival in patients with non‐metastatic clear cell renal cell carcinoma (ccRCC) compared with a Cox proportional hazards (CPH) approach.

**Patients and Methods:**

Patients undergoing curative surgery for non‐metastatic ccRCC between 2010 and 2018 were identified from the DaRenCa Study‐3, a nationwide register‐based cohort study. Three recurrence prediction models were developed: an extreme gradient boosting (XGBoost) model, a feature‐matched CPH model and a pathology‐based CPH model. The data set was divided into training and test cohorts. Missing data were addressed using multiple imputation for the CPH models, whereas XGBoost handled missing values inherently. Model performance was evaluated using the concordance index (C‐index) with 1000 bootstrap resamples. The XGBoost model was also compared with the Leibovich nomogram.

**Results:**

Among 2782 patients, with a median follow‐up of 7.3 years, 13.7% developed a recurrence. In the test cohort, the XGBoost model showed higher discrimination than both CPH models. Compared with the best performing pathology‐based CPH model, XGBoost demonstrated a paired bootstrap difference in Uno's C‐index of 0.022 (95% CI 0.005–0.038). The model also identified a subgroup of patients with a very low risk of recurrence (<3% after 10 years) and demonstrated improved clinical risk stratification, with clearer separation between risk groups, higher hazard ratios between groups and larger differences in 5‐year recurrence‐free survival compared with established models. This improved risk stratification could reduce follow‐up imaging by approximately 11% compared with current EAU guideline recommendations. Limitations include the retrospective design and lack of external validation.

**Conclusion:**

The XGBoost model provided improved prediction of recurrence compared with CPH models and the Leibovich nomogram, supporting more precise risk stratification. With external validation, this approach may help reduce unnecessary imaging after surgery.

## 1 INTRODUCTION

The incidence of renal cell carcinoma (RCC) is increasing, now accounting for approximately 3% of all malignancies, and around 85% of Danish patients present with non‐metastatic disease.^1^, ^2^ Following curative‐intent surgery, up to 30% of patients with non‐metastatic RCC will eventually develop metastatic disease, with risk varying according to pathological features.^3^ The European Association of Urology (EAU) Guidelines recommend risk stratification based on the Leibovich 2003 nomogram for scheduling postoperative follow‐up after surgery in clear cell (cc)RCC patients.^4^ The Leibovich 2003 model is based on a multivariable Cox proportional hazards (CPH) regression framework,^5^ which, while interpretable, has limitations such as the inability to model nonlinearities and interaction effects.^6^ Moreover, the nomogram demonstrated lower predictive performance in a previous Danish cohort compared with that reported in the original study.^7^

Accurate estimation of recurrence risk is essential for optimizing postsurgical follow‐up intensity and for minimizing unnecessary imaging.^8^ One way to improve postsurgical follow‐up could be by implementing a model capable of better differentiating between patients with low and high risk of recurrence. Flexible machine‐learning approaches, such as extreme gradient boosting (XGBoost),^9^ may offer such potential. The XGBoost model can implicitly capture non‐linearities and interaction effects,^6^ potentially reflecting the underlying biological heterogeneity of ccRCC more accurately.^10^

Against this background, we sought to develop an XGBoost survival model for better assessing recurrence‐free survival (RFS) in patients with ccRCC following curative‐intent surgery and to compare its performance with two CPH‐based models: a pathology‐based CPH model reflecting established prognostic factors and a feature‐matched CPH model using the same predictors as the XGBoost model. Moreover, we hypothesized that the XGBoost model would outperform the traditional CPH‐based Leibovich 2003 nomogram^5^ in stratifying recurrence risk, owing to its ability to capture non‐linear effects and leverage informative missingness.

## 2 MATERIAL AND METHODS

The study population was identified from the Danish nationwide DaRenCa Study‐3,^11^ which includes all patients diagnosed with histologically or cytologically confirmed RCC in Denmark between 2010 and 2018. The database integrates data from multiple national health registries (DaRenCa,^12^ Civil Registration System,^13^ National Patient Register,^14^ Danish Pathology Register,^15^ Danish Cancer Register^16^ and the Danish Cause of Death Register^17^), supplemented by manually extracted information from individual patient medical records. Patients with metastatic disease at diagnosis, as determined by routine clinical staging, including contrast‐enhanced CT of the thorax and abdomen, or identification of metastasis within 120 days after surgery, were excluded (*n* = 941), as were individuals with ≥12 missing variables (*n* = 150). We included 2782 patients who were diagnosed with non‐metastatic ccRCC between August 2010 and August 2018 and underwent curative‐intent surgery.

RFS was defined as the time from the date of surgery to the date of either local or distant recurrence, with patients censored at last follow‐up or death without recurrence.

The Danish Health Data Board granted ethical and legal approval for the study in accordance with Danish law (journal number 3‐3013‐2902/1).

### 2.1 Statistics

Potential predictors were selected a priori and included demographic and clinical characteristics, laboratory markers, surgery‐related factors and pathological features. Demographic and clinical variables comprised region and year of surgery, age, sex, tumour side, BMI, smoking status, hypertension, presenting symptoms, performance status and ASA score. Laboratory markers included haemoglobin and neutrophil count. Surgery‐related variables encompassed preoperative biopsy, time to surgery and surgical approach. Pathological variables included pT and pN stage, Fuhrman grade, tumour necrosis, sarcomatoid and rhabdoid differentiation, tumour size and resection margin status.

The data set was randomly divided into a training set (*n* = 2225) and a test set (*n* = 557) using an 80/20 split, stratified by recurrence rate. Missing values were imputed using the Multiple Imputations by Chained Equations (MICE) algorithm with five iterations on the training set, and the resulting imputation model was applied to the test set to avoid data leakage. Categorical variables were one‐hot encoded, and numerical features were standardized using the scaling parameters derived from the training set. MICE imputation, one‐hot encoding and feature scaling were not applied to the XGBoost model as the XGBoost algorithm inherently handles missing and categorical data.^9^

**FIGURE 1:**
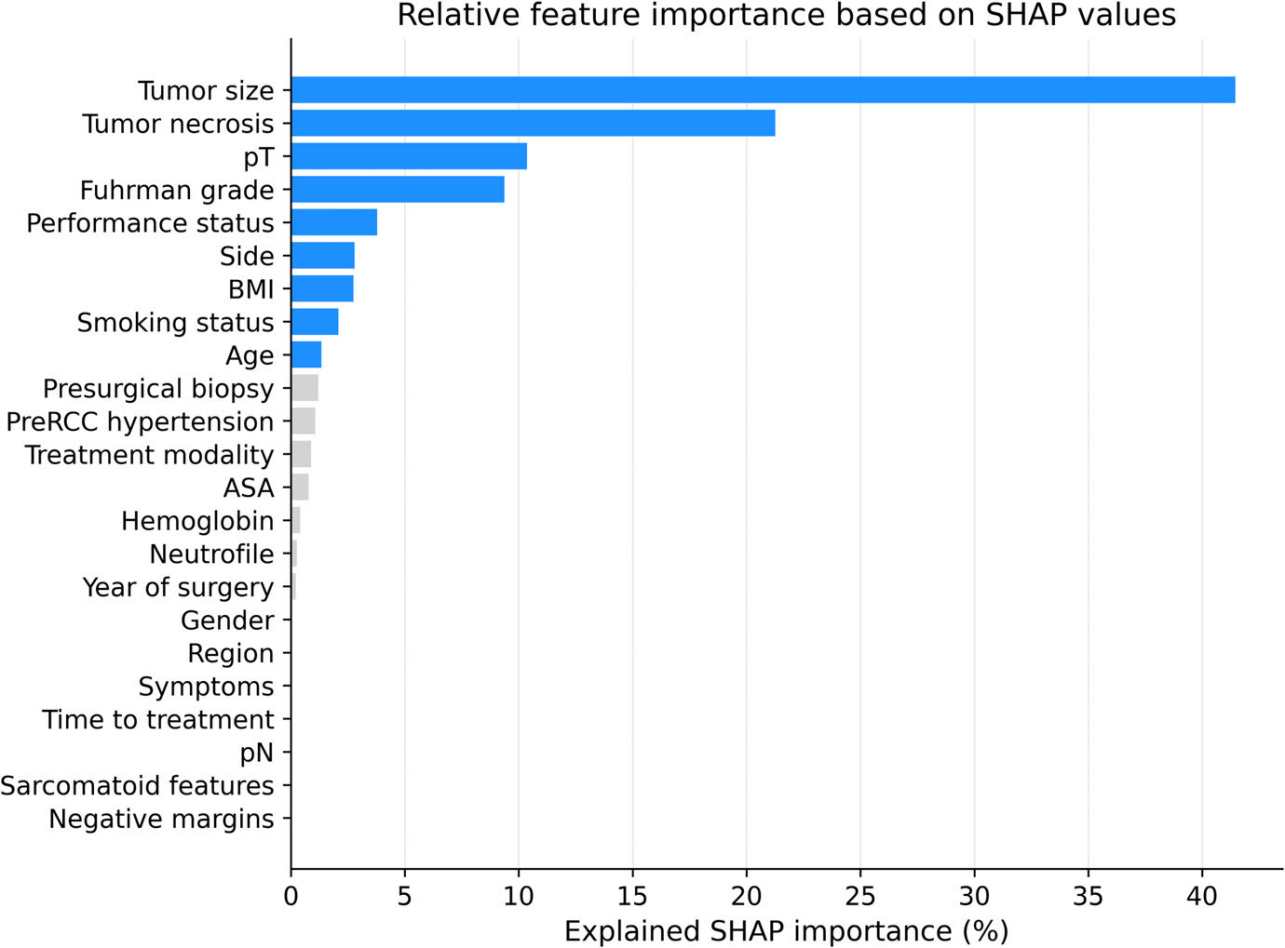
Percentage of the absolute SHAP importance explained by each variable with the final nine variables coloured blue, as they explain ≥95% of the cumulative SHAP importance. SHAP, Shapley additive explanations.

Three models were developed and compared: an XGBoost survival model, a pathology‐based CPH model (CPH‐Path) and a feature‐matched CPH model (CPH‐Match). Predictors for the XGBoost model were selected using Shapley Additive Explanation (SHAP) values,^18^ retaining variables that together explained ≥95% of the cumulative SHAP importance in the training set (Figure 1). Hyperparameters were optimized using tenfold cross‐validation within the training set (Table S1). The CPH‐Path model included established pathological predictors (tumour size, necrosis, pT stage and Fuhrman grade). Tumour size was modelled on the log scale to account for previously described non‐linear associations with recurrence risk. The CPH‐Match model used the same predictors as the final XGBoost model to allow direct comparison between modelling approaches. Hazard ratios for the CPH‐Path and CPH‐Match models are reported in Table S2.

Model discrimination was evaluated using Uno's concordance index (C‐index) and the integrated AUC (iAUC), and calibration using the integrated Brier score (IBS) across a 0.5–5‐year prediction horizon. Model performance was estimated through 1000 bootstrap resamples, reporting mean values and 95% confidence intervals (CIs). Variable selection in the CPH models and hyperparameters for the XGBoost model were held constant across all bootstrap iterations.

### 2.2 Risk stratification

For the updated risk stratification, we defined a group with very low risk of recurrence using a hazard ratio (HR) threshold that encompassed fewer than 2% of recurrence events across five cross‐validation folds in the training set. The final threshold was determined as the median HR value across the five folds. We further defined groups with low, intermediate and high risk of recurrence using two additional HR thresholds, selected to maximize the minimum log‐rank separation between adjacent Kaplan–Meier curves (i.e., low vs. intermediate risk and intermediate vs. high risk) across the fivefold cross‐validation procedure. For comparison with the guideline‐recommended Leibovich nomogram,^5^ currently used in the clinic,^4^ HRs were computed for each patient using the multivariable CPH model. In addition, the GRANT score^19^ and the American Urological Association (AUA) guideline‐based postoperative risk groups for comparison, classifying patients as low, intermediate, high or very high risk^20^ were calculated as a contemporary simplified prognostic model to allow comparison with a more recent clinical risk stratification approach. As macroscopic positive surgical margin status could not be reliably identified in the data set, this criterion was not included in the AUA classification.

Other established prognostic tools such as Leibovich^21^ and ASSURE^22^ were considered; however, these require detailed anatomical descriptors not available in the data set and could not be reliably inferred from pT stage alone. Analyses were therefore restricted to models that could be implemented without approximation.

Statistical analyses were performed using **Python version 3.12.2** (Python Software Foundation).

## 3 RESULTS

Baseline characteristics of the 2782 patients are presented in Table 1. The median follow‐up was 7.3 (IQR 5.5–9.5) years. In total, 13.7% of patients experience a recurrence, of which 17.9% were local and 82.1% were metastatic.

**TABLE 1:** Baseline characteristics of 3288 patients surgically treated for non‐metastatic ccRCC stratified on training and test set.

Variable	Training set (*N* = 2225)	Testing set (*N* = 557)	Missing (%)
Region of Denmark, *n* (%)			0/0
Capital	580 (26.1)	132 (23.7)	
Central	424 (19.1)	116 (20.8)	
North	299 (13.4)	67 (12.0)	
Zealand	419 (18.8)	96 (17.2)	
South	503 (22.6)	146 (26.2)	
Year of surgery, median (IQR)	2015 (2013–2016)	2014 (2013–2016)	0/0
Age, median (IQR)	65.9 (57.5–72.2)	64.2 (55.9–71.6)	0/0
Male, *n* (%)	1429 (64.2)	358 (64.3)	0/0
Side, *n* (%)			34.3/34.1
Bilateral	17 (0.8)	5 (0.9)	
Right	711 (32.0)	176 (31.6)	
Left	733 (32.9)	186 (33.4)	
Missing	764 (34.3)	190 (34.1)	34.3/34.1
BMI, median (IQR)	27.1 (24.1–30.8)	26.9 (24.5–30.0)	40.3/42.5
Smoking			37.5/38.6
Never smoked	547 (24.6)	140 (25.1)	
Former smoker	482 (21.7)	101 (18.1)	
Smoker	362 (16.3)	101 (18.1)	
Missing	834 (37.5)	215 (38.6)	37.5/38.6
PreRCC hypertension, *n* (%)	779 (35.0)	192 (34.5)	36.8/35.5
Symptoms at diagnosis, *n* (%)	1135 (51.0)	298 (53.5)	0/0
Performance status, *n* (%)			37.5/37.2
0	968 (43.5)	255 (45.8)	
1	336 (15.1)	72 (12.9)	
2	65 (2.9)	17 (3.1)	
3	15 (0.7)	6 (1.1)	
4	7 (0.3)	0 (0.0)	
Missing	834 (37.5)	207 (37.2)	37.5/37.2
ASA score, *n* (%)			53.3/53.3
1	192 (8.6)	46 (8.3)	
2	603 (27.1)	156 (28.0)	
3	238 (10.7)	56 (10.1)	
4	7 (0.3)	2 (0.4)	
Missing	1185 (53.3)	297 (53.3)	53.3/53.3
Laboratory markers			
Haemoglobin, mmol/L, median (IQR)	8.0 (7.3–9.0)	8.0 (7.7–9.0)	7.1/7.9
Neutrophil count, ×10^9^/L, median (IQR)	4.6 (3.3–6.0)	4.2 (3.0–6.0)	18.9/17.1
Surgery‐related factors, *n* (%)			
Preoperative biopsy performed	494 (22.2)	113 (20.3)	34.4/34.6
Laparoscopic radical nephrectomy	1139 (51.2)	284 (51.0)	
Open radical nephrectomy	300 (13.5)	77 (13.8)	
Laparoscopic partial nephrectomy (including robot‐assisted)	494 (22.2)	111 (19.9)	
Open partial nephrectomy	232 (10.4)	60 (10.8)	
Missing procedure information	60 (2.7)	25 (4.5)	2.7/4.5
Time to surgery, median (IQR)	0.0 (0.0–18.0)	0.0 (0.0–17.0)	0/0
Pathological features, *n* (%)			
Tumour size, mm, median (IQR)	47 (31–72)	48 (32–71)	3.9/4.5
T1a	535 (24.0)	126 (22.6)	
T1b	549 (24.7)	158 (28.4)	
T2	254 (11.4)	65 (11.7)	
T3a	543 (24.4)	129 (23.2)	
T3b	36 (1.6)	8 (1.4)	
Missing	308 (13.8)	71 (12.7)	13.8/12.7
pN1, *n* (%)	11 (0.5)	4 (0.7)	0/0
Fuhrman grade, n (%)			12.7/13.6
1	302 (13.6)	82 (14.7)	
2	1086 (48.8)	262 (47.0)	
3	411 (18.5)	111 (19.9)	
4	143 (6.4)	26 (4.7)	
Missing	283 (12.7)	76 (13.6)	12.7/13.6
Tumour necrosis, *n* (%)	442 (19.9)	101 (18.1)	0/0
Sarcomatoid differentiation, *n* (%)	54 (2.4)	9 (1.6)	0/0
Resection margins, *n* (%)			0/0
Negative	2108 (94.7)	532 (95.5)	
Positive	117 (5.3)	25 (4.5)	

Abbreviations: ASA, American Society of Anesthesiologists; BMI, Body Mass Index; ccRCC, clear cell renal cell carcinoma; IQR, Interquartile Range.

Model performance metrics (C‐index, iAUC and IBS) based on 1000 stratified bootstrap resamples are summarized in Table 2. XGBoost showed higher discrimination than both CPH models, with a statistically significant paired bootstrap difference of 0.022 versus CPH‐Path (95% CI 0.005–0.038), outperforming it in 99.5% of resamples. Brier scores were similar across time points, although variability was greater for XGBoost (Figure S1).

**TABLE 2:** Bootstrapped estimates of Uno's C‐index, iAUC and IBS for hyperparameter‐optimized XGBoost survival model and CPH‐based models in the training and test cohorts.

Model	Uno's C train	Uno's C test	IBS train	IBS test	iAUC train	iAUC test
CPH‐Path*	0.77 (0.74–0.80)	0.77 (0.76–0.78)	0.062 (0.058–0.065)	0.063 (0.062–0.064)	0.85 (0.83–0.88)	0.81 (0.80–0.82)
CPH‐Match**	0.77 (0.79–0.80)	0.76 (0.75–0.78)	0.061 (0.057–0.065)	0.064 (0.062–0.065)	0.86 (0.83–0.88)	0.80 (0.79–0.82)
XGBoost***	0.81 (0.79–0.83)	0.79 (0.78–0.81)	0.061 (0.057–0.065)	0.064 (0.062–0.067)	0.86 (0.84–0.89)	0.80 (0.79–0.82)

*Note*: Metrics are based on 1000 bootstrap repetitions, with iAUC and IBS calculated over the 0.5–5‐year time horizon.

Abbreviations: C‐index, concordance index; CPH, Cox proportional hazards; iAUC, integrated area under curve; IBS, integrated Brier score; XGBoost, Extreme Gradient Boosting.

* Variables included: tumour size, tumour necrosis, pT stage, Fuhrman grade.

** Variables included: tumour size, tumour necrosis, pT stage, Fuhrman grade, Age, BMI, PS, Smoking status, Side**.

*** Optimal hyperparameters: Number of trees = 110, tree depth = 9, minimum child weight = 27, learning rate = 0.03, subsample (patients per tree) = 65%, subsamples (variables per tree) = 99%, *λ* = 16.1, *γ* = 3.7, *α* = 11.3.

**FIGURE 2:**
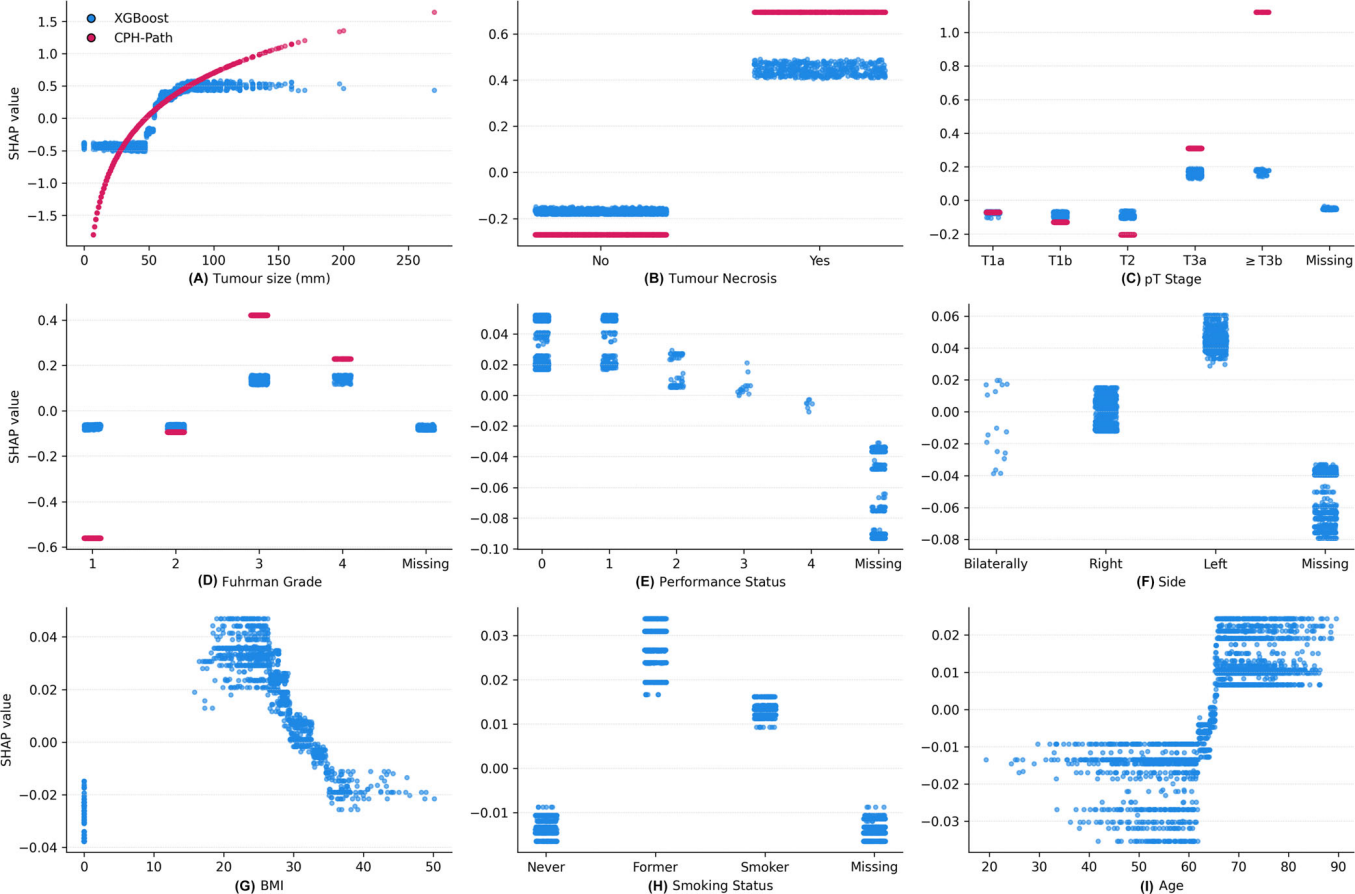
SHAP dependence plots for the XGBoost model (blue) compared with the CPH‐path model (red). Panels show the relationship between feature value and SHAP contribution for (A) tumour size, (B) tumour necrosis, (C) pT stage, (D) Fuhrman grade, (E) performance status, (F) side, (G) BMI, (H) smoking status and (I) age. CPH‐path, pathological Cox proportional hazards; SHAP, Shapley additive explanations; XGBoost, extreme gradient boosting.

Tumour‐related variables (tumour size, necrosis, pT stage and Fuhrman grade) contributed most to XGBoost model predictions, whereas patient‐related variables (age, BMI, smoking status and performance status) had less influence (Figure 1). Higher tumour size, pT stage, grade and necrosis were associated with increased recurrence risk. SHAP analyses showed non‐linear effects for tumour size, BMI and age (Figure 2). In the test set, XGBoost also outperformed the 2003 Leibovich nomogram (Uno's C‐index 0.784 vs. 0.740).

Decision curve analysis for 3‐year RFS found that both the XGBoost and CPH‐Path models outperformed treat‐all and treat‐none strategies across a range of thresholds: Figure S2. XGBoost showed slightly higher net benefit at thresholds of 0.2–0.5, whereas CPH‐Path performed better at 0.1–0.2.

**FIGURE 3:**
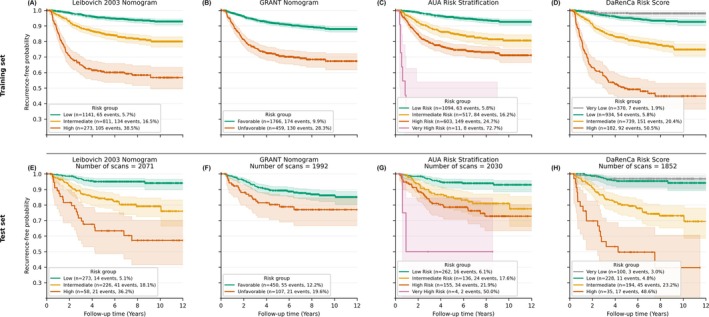
Kaplan–Meier curves showing recurrence‐free survival stratified by risk groups for the Leibovich 2003 nomogram, the GRANT nomogram and the XGBoost‐based DaRenCa risk score in both the training and independent test sets. (A–C) training set: (A) Leibovich 2003 nomogram, (B) GRANT nomogram and (C) DaRenCa risk score. (D–F) independent test set: (D) Leibovich 2003 nomogram, (E) GRANT nomogram and (F) DaRenCa risk score. For the DaRenCa risk score, the very low‐risk group was defined to minimize the number of recurrences, while the remaining low‐, intermediate‐ and high‐risk groups were derived by maximizing the minimum pairwise log‐rank separation between curves. XGBoost, extreme gradient boosting.

Figure 3 shows risk‐stratified Kaplan–Meier curves for the Leibovich nomogram and the XGBoost model in both the training and independent test sets. The XGBoost model identified a distinct very low‐risk group and smaller intermediate‐ and high‐risk groups with a higher concentration of recurrence events compared with the 2003 Leibovich nomogram and the AUA classification. Based on EAU^4^ and AUA guidelines,^20^ and assuming two scans for patients in the very low‐risk group, this corresponds to an estimated 10.6% and 8.8% reduction in follow‐up imaging during the first three postoperative years.

Table 3 summarizes the distribution of patients across risk groups, 5‐year RFS and HRs for each model in the independent test cohort. The DaRenCa risk score identified a very low‐risk group with a recurrence rate of 3.0% and a 5‐year RFS of 96.9%, compared with 5.1% and 95.1% for the low‐risk group in the Leibovich model, 6.1% and 94.5% for the low‐risk group in the AUA classification and 12.2% and 89.3% for the favourable group in the GRANT model. At the high‐risk end, the DaRenCa risk score showed a higher recurrence rate (48.6%) and lower 5‐year RFS (49.6%) than the Leibovich high‐risk group (36.2% and 63.4%) and the AUA high‐risk group (21.9% and 78.6%), indicating greater separation between prognostic groups. The AUA very‐high‐risk group had a recurrence rate of 50.0% and a 5‐year RFS of 50.0%; however, it only included four patients. HRs between risk groups were also larger for the DaRenCa risk score, reflecting a steeper gradient in recurrence risk across categories. Although separation between the very low‐ and low‐risk groups was limited, both groups consistently showed low recurrence risk.

**TABLE 3:** Risk group distribution, number of recurrences, 5‐year recurrence‐free survival and hazard ratios in the independent test cohort for the Leibovich 2003, GRANT, AUA and DaRenCa risk models.

Model	Risk group	*n*	Recurrences *n* (%)	5‐year RFS % (95% CI)	HR (95% CI)	*p*‐value
Leibovich 2003	Low	273	14 (5.1%)	95.1 (91.7–97.1)	Reference	
	Intermediate	226	41 (18.1%)	84.2 (78.5–88.5)	4.06 (2.21–7.46)	<0.001
	High	58	21 (36.2%)	63.4 (48.7–75.0)	9.71 (4.93–19.10)	<0.001
GRANT	Favourable	450	55 (12.2%)	89.3 (86.0–91.9)	Reference	
	Unfavourable	107	21 (19.6%)	80.2 (70.7–86.9)	1.96 (1.19–3.25)	0.009
AUA	Low	262	16 (6.1%)	94.5 (90.9–96.7)	Reference	
	Intermediate	136	24 (17.6%)	85.1 (77.6–90.2)	3.16 (1.68–5.94)	<0.001
	High	155	34 (21.9%)	78.6 (70.8–84.6)	4.49 (2.48–8.15)	<0.001
	Very High	4	2 (50.0%)	50.0 (5.8–84.5)	17.01 (3.90–74.26)	<0.001
DaRenCa Risk Score	Very Low	100	3 (3.0%)	96.9 (90.6–99.0)	Reference	
	Low	228	11 (4.8%)	95.4 (91.5–97.5)	1.68 (0.47–6.01)	0.428
	Intermediate	194	45 (23.2%)	80.1 (73.5–85.2)	9.36 (2.91–30.14)	<0.001
	High	35	17 (48.6%)	49.6 (31.3–65.6)	28.72 (8.38–98.36)	<0.001

*Note*: Hazard ratios were estimated using univariate Cox proportional hazards models with the lowest risk group as reference. 5‐year recurrence‐free survival (RFS) estimates were derived from Kaplan–Meier analyses, with 95% confidence intervals (CIs). Recurrence rates are presented as the number and percentage of patients experiencing recurrence within each risk group.

Abbreviations: AUA, American Urological Association; CI, confidence interval; HR, hazard ratio; RFS, recurrence‐free survival.

Figure S3 presents SHAP dependency plots for the XGBoost model in the independent test cohort, with observations coloured by predicted risk group. Patients with the same pT stage or Fuhrman grade were often assigned to different risk groups depending on other variables, illustrating that the model considers multiple factors when estimating recurrence risk.

## 4 DISCUSSION

In this nationwide registry‐based study of patients undergoing curative‐intent surgery for non‐metastatic ccRCC, the XGBoost model demonstrated improved discrimination compared with CPH‐based models and established prognostic tools. While improvements in global discrimination were modest, the model provided clearer separation between risk groups compared with the Leibovich 2003, GRANT and AUA guideline‐based classifications. SHAP analyses indicated that tumour‐related factors—tumour size, necrosis, pT stage and Fuhrman grade—were the main determinants of recurrence risk. Compared with CPH models, XGBoost better captured non‐linear relationships and incorporated missingness as an informative feature. The model also outperformed the 2003 Leibovich nomogram and identified a subgroup of patients at very low risk of recurrence (<3%).

In addition, it defined smaller intermediate‐ and high‐risk groups, which could reduce follow‐up imaging by approximately 11% within the first three postoperative years compared with current European guidelines^4^ and by approximately 9% compared with current American guidelines.^20^ Importantly, the DaRenCa risk score identified a subgroup with very low recurrence risk (3.0% recurrence; 5‐year RFS 96.9%), which was slightly lower than the low‐risk group defined by the Leibovich model (5.1%; 95.1%). At the high‐risk end, the DaRenCa risk score concentrated recurrence events within a smaller group, with a higher recurrence rate and lower 5‐year RFS compared with both the Leibovich and GRANT scores. Although the AUA very‐high‐risk group had a comparable recurrence rate, it included only four patients in the test set and should therefore be interpreted with caution. Thus, the DaRenCa risk score were the best model to separate between the lowest and highest recurrence risk groups.

This study has several limitations. First, the retrospective design may have introduced residual confounding despite the use of comprehensive nationwide registry data. Second, although internal validation was performed, external validation is required, and the generalizability to non‐Danish populations remains uncertain. In addition, recurrence detection relied on routine clinical follow‐up, which may vary across institutions despite national recommendations. Furthermore, recurrence risk alone may not fully determine the optimal follow‐up strategy, as the benefit of surveillance depends on factors such as timing and pattern of recurrence, including whether recurrence is amenable to treatment. In addition, the cohort consisted exclusively of patients treated in the pre‐adjuvant therapy era, and the model therefore reflects outcomes in this setting; its performance in patients receiving other treatment modalities, including ablative or adjuvant therapies, remains to be established. Fuhrman grade, although available in this data set, has largely been replaced by the ISUP/WHO grading system in current practice, which may limit applicability to more recent cohorts.^23^ While both systems are based on nuclear features, Fuhrman uses multiple criteria, whereas ISUP/WHO mainly emphasizes nucleolar prominence.^24^ This may result in downgrading, particularly from Fuhrman Grade 2 to ISUP Grade 1, without evident clinical impact. Given the good correlation between the systems,^25^ the findings remain clinically relevant. Finally, the models were based on routinely collected clinical and pathological variables; inclusion of molecular biomarkers or imaging‐derived features may improve predictive performance. Death without recurrence was treated as a censoring event rather than a competing risk, which may influence absolute risk estimates, although this approach aligns with standard RFS modelling and allows direct comparison with established prognostic models.

The study also has important strengths. It is based on high‐quality nationwide Danish registry data, enabled by the unique personal identification number, allowing complete linkage and detailed review of patient records across the country. Baseline characteristics were similar between the training and test cohorts across all variables, supporting the validity of the model evaluation. External validation is required before clinical implementation.

The use of informative missingness represents both a strength and a limitation of the XGBoost model. Unlike traditional imputation approaches, which may dilute information contained in missing values, tree‐based methods can retain and use this information, potentially improving predictive performance. This is illustrated in Figure 2, where missing tumour side was associated with lower SHAP contributions and lower predicted recurrence risk. A corresponding chi‐square test showed a lower proportion of recurrence events among patients with missing tumour side (*p* < 0.001).

However, such patterns may reflect underlying clinical or administrative processes rather than true biological effects. The model may therefore capture aspects of clinical decision‐making or data recording practices, which can vary across healthcare systems and introduce bias. These findings highlight the need for external validation to ensure generalizability.

Unlike the log‐linear assumptions of the CPH framework underlying the Leibovich nomogram, XGBoost captured complex relationships between predictors and outcomes, which may better reflect tumour biology. In SHAP analyses, the association between tumour size and recurrence followed a sigmoid pattern, consistent with previous reports of a plateau effect in larger tumours, possibly related to necrosis or indolent behaviour.^26^ pT stage contributed less once tumour size was included, which is expected given that pT1–pT2 categories are defined by tumour size. Accordingly, these stages showed similar SHAP contributions, whereas ≥pT3 stages had higher contributions, reflecting additional anatomical features such as venous or perinephric invasion. This is consistent with the updated 2018 Leibovich model and a CPH model based on prospective data from 2021 (ASSURE trial), both of which replaced pT stage with anatomical tumour location.^21^, ^22^ Further patterns were observed in the SHAP analyses (Figure 2). Fuhrman grade separated clearly into low‐grade (1–2) and high‐grade (3–4) tumours, in line with prior evidence supporting a simplified two‐tier grading system.^27^ An inverse association between BMI and recurrence risk was also observed, consistent with the “obesity paradox” described in RCC.^28^ Importantly, SHAP plots (Figure S3) showed that patients within the same category of a single predictor were distributed across different risk groups. For example, patients with the same pT stage or Fuhrman grade had varying contributions depending on other variables, such as tumour size or necrosis. This indicates that the model integrates multiple predictors when estimating recurrence risk. Such interactions are captured by gradient boosting methods but are difficult to model within the CPH framework without specifying interaction terms. This likely contributes to the improved risk stratification observed in the Kaplan–Meier analysis (Figure 3).

The continuous risk estimates generated by XGBoost allowed more precise risk stratification and threshold definition than CPH‐based clinical scoring systems, such as the Leibovich and the ASSURE trial 2021 model, which rely on categorization of predictors.^5^, ^22^ However, this increased flexibility comes at the cost of reduced transparency compared with traditional CPH models. Although SHAP‐based visualizations improve interpretability,^18^, ^29^ they are post hoc and may reflect features of the model rather than underlying biology. The complexity of XGBoost also limits translation into a simple point‐based score. This may be addressed through implementation as a web‐based calculator, enabling clinical use without manual calculations. Despite improved discrimination, calibration was less consistent, as reflected by wider IBS confidence intervals compared with the CPH model. These findings highlight the need for cautious interpretation of individual SHAP outputs and for validation in independent cohorts to confirm generalizability.

Although the XGBoost model showed improved discrimination compared with CPH‐based models, the magnitude of this improvement was modest. This suggests that current models may be approaching the limits of predictive performance based on routinely available clinical and pathological variables. Across modelling approaches, the most influential predictors remained tumour size, pT stage, tumour necrosis and nuclear grade, indicating that these factors capture the majority of prognostic information available in this setting. Further gains in predictive accuracy are therefore likely to depend on the incorporation of novel tumour‐specific data, such as molecular or imaging‐derived biomarkers. Despite this, the use of machine learning allowed more refined risk stratification, with clearer separation of patient groups. This has potential clinical implications, as improved stratification may support more tailored follow‐up strategies, including more efficient use of imaging and healthcare resources.

## 5 CONCLUSION

This study demonstrated the potential of machine‐learning–based survival modelling to enhance postoperative risk stratification in patients with non‐metastatic ccRCC. By improving prognostic precision and enabling more individualized follow‐up strategies, such approaches could support more cost‐efficient and patient‐centred care. Validation in independent cohorts remained essential before clinical implementation.

## AUTHOR CONTRIBUTIONS

Conceptualization, study design, data analysis, data interpretation and manuscript drafting were performed by PH, RDP and FFT. Data acquisition was performed by HA‐H, EH, ML, ST, RB and LL. Statistical analyses were performed by PH. All authors critically revised the manuscript, approved the final version and agree to be accountable for all aspects of the work.

## CONFLICT OF INTEREST STATEMENT

The authors declare no conflicts of interest.

## Supporting information


**Table S1.** Hyperparameter optimization search parameters for XGBoost.

**Table S2.** Hazard ratios for the CPH‐models.

**Figure S1:** Time‐dependent Brier scores (mean ± standard deviation) for the XGBoost and CPH models across the 0.5–5‐year prediction. Abbreviations: XGBoost: Extreme Gradient Boosting, CPH‐Path: pathology‐based Cox Proportional Hazards.

**Figure S2:** Decision curve analysis curve showing the net benefit at different thresholds at three years for the XGBoost model compared to the CPH‐Path model against the treat all and treat non options. Abbreviations: CPH‐Path: pathology‐based Cox Proportional Hazards, XGBoost: Extreme Gradient Boosting.

**Figure S3:** SHAP dependence plots for the XGBoost model shown on the independent test set, with patients coloured by their risk group: Very Low (grey), Low (green), Intermediate (yellow), High (red). Abbreviations: XGBoost: Extreme Gradient Boosting, SHAP: Shapley Additive Explanations.
