# Supplementary material for: DaRenCa risk score: A prognostic model for recurrence in clear cell renal cell carcinoma
Source: BJUI Compass. 2026 Jun 3;7(6):e70234. doi: 10.1002/bco2.70234 (PMC13240388; doi:10.1002/bco2.70234)
Supplement: Supplementary file 1 — Table S1. Hyperparameter optimization search parameters for XGBoost. [file BCO2-7-e70234-s002.docx]

| **Supplementary Table 1** Hyperparameter optimization search parameters for XGBoost | | | |
| --- | --- | --- | --- |
| **Hyperparameter** | **Description (Plain Language)** | **Search Space / Values Tested** | **Optimal Hyperparameter** |
| Number of trees | Number of boosting rounds | 100-500 | 110 |
| Max tree depth | Limits the number of sequential decisions a single tree can make. | 3-10 | 9 |
| Learning rate | Step size in boosting | 0.010-0.200 | 0.029 |
| Minimum child weight | Minimum sample weight needed to create a split | 5-30 | 27 |
| Subsample | Fraction of patients used per tree | 0.60-1.00 | 0.65 |
| Column subsampling (per tree) | Fraction of variables used per tree | 0.60-1.00 | 0.99 |
| Gamma | Minimum loss reduction required for a split | 1.00-10.00 | 3.71 |
| Lambda (L2 regularization) | L2 regularization strength | 0.00-20.00 | 16.08 |
| Alpha (L1 regularization) | L1 regularization strength | 0.00-20.00 | 11.27 |
