# Supplementary material for: DaRenCa risk score: A prognostic model for recurrence in clear cell renal cell carcinoma
Source: BJUI Compass. 2026 Jun 3;7(6):e70234. doi: 10.1002/bco2.70234 (PMC13240388; doi:10.1002/bco2.70234)
Supplement: Supplementary file 2 — Table S2. Hazard ratios for the CPH‐models. [file BCO2-7-e70234-s005.docx]

| Supplementary Table 2, Hazard ratios for the CPH-models | | | |
| --- | --- | --- | --- |
| **Supplementary Table 2a: Hazard ratios for CPH-Path** | | | |
| Covariate | HR | 95% CI | p-value |
| Tumour size, log-transformed | 1.74 | 1.39-2.17 | <0.005 |
| Tumour necrosis | 2.62 | 2.03-3.38 | <0.005 |
| pT-stage |  |  |  |
| T1a | Ref |  |  |
| T1b | 0.94 | 0.59-1.50 | 0.80 |
| T2 | 0.88 | 0.48-1.60 | 0.67 |
| T3a | 1.46 | 0.87-2.47 | 0.15 |
| ≥T3b | 3.29 | 1.62-6.69 | <0.005 |
| Fuhrman grade |  |  |  |
| 1 | Ref |  |  |
| 2 | 1.60 | 0.94-2.70 | 0.08 |
| 3 | 2.67 | 1.54-4.62 | <0.005 |
| 4 | 2.20 | 1.21-4.00 | 0.01 |
| **Supplementary Table 2b: Hazard ratios for CPH-Match** | | | |
| Covariate | HR | 95% CI | p-value |
| Tumour size, log-transformed | 1.76 | 1.41-2.20 | <0.005 |
| Tumour necrosis | 2.59 | 2.01-3.34 | <0.005 |
| pT-stage |  |  |  |
| T1a | Ref |  |  |
| T1b | 0.94 | 0.59-1.49 | 0.35 |
| T2 | 0.87 | 0.47-1.59 | 0.63 |
| T3a | 1.44 | 0.85-2.42 | 0.18 |
| ≥T3b | 3.12 | 1.53-6.37 | <0.005 |
| Fuhrman grade |  |  |  |
| 1 | Ref |  |  |
| 2 | 1.57 | 0.93-2.65 | 0.09 |
| 3 | 2.63 | 1.52-4.56 | <0.005 |
| 4 | 2.16 | 1.18-3.93 | 0.01 |
| Age | 1.07 | 0.94-1.22 | 0.29 |
| BMI | 0.97 | 0.86-1.10 | 0.67 |
| Performance status |  |  |  |
| 0 | Ref |  |  |
| 1 | 1.05 | 0.80-1.36 | 0.74 |
| ≥2 | 0.97 | 0.59-1.61 | 0.92 |
| Smoking |  |  |  |
| Never smoked | Ref |  |  |
| Former smoker | 1.17 | 0.91-1.52 | 0.22 |
| Smoker | 1.06 | 0.79-1.44 | 0.69 |
| Side |  |  |  |
| Left |  |  |  |
| Right | 1.16 | 0.93-1.46 | 0.19 |
| Abbreviations: CPH: Cox proportional hazards, HR: Hazard ratio, CI: Confidence interval, BMI: Body mass index | | | |
