# Supplementary figures and images for: DaRenCa risk score: A prognostic model for recurrence in clear cell renal cell carcinoma
Source: BJUI Compass. 2026 Jun 3;7(6):e70234. doi: 10.1002/bco2.70234 (PMC13240388; doi:10.1002/bco2.70234)

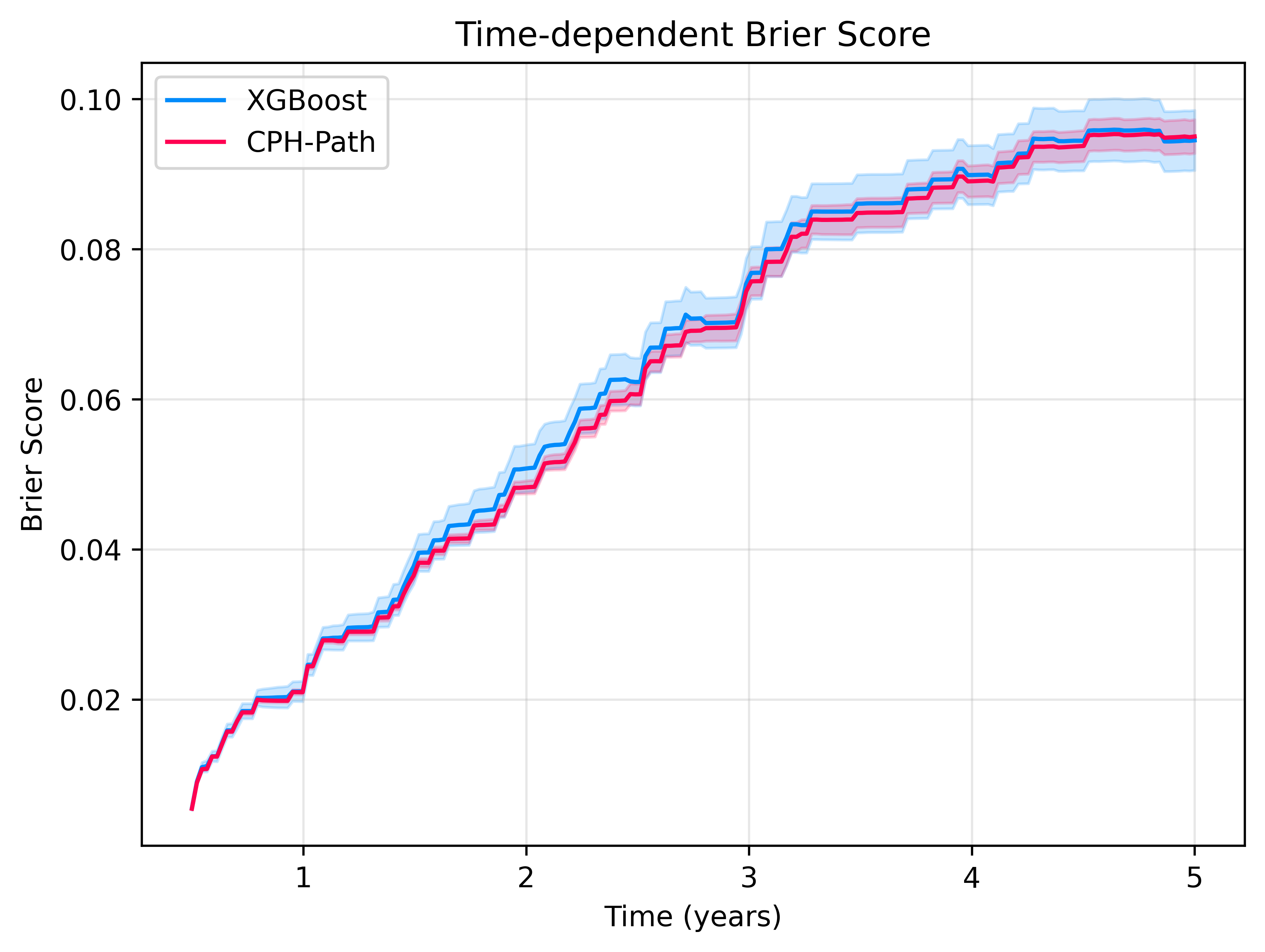

Supplement: Supplementary file 3 — Figure S1: Time‐dependent Brier scores (mean ± standard deviation) for the XGBoost and CPH models across the 0.5–5‐year prediction. Abbreviations: XGBoost: Extreme Gradient Boosting, CPH‐Path: pathology‐based Cox Proportional Hazards. [file BCO2-7-e70234-s003.png]

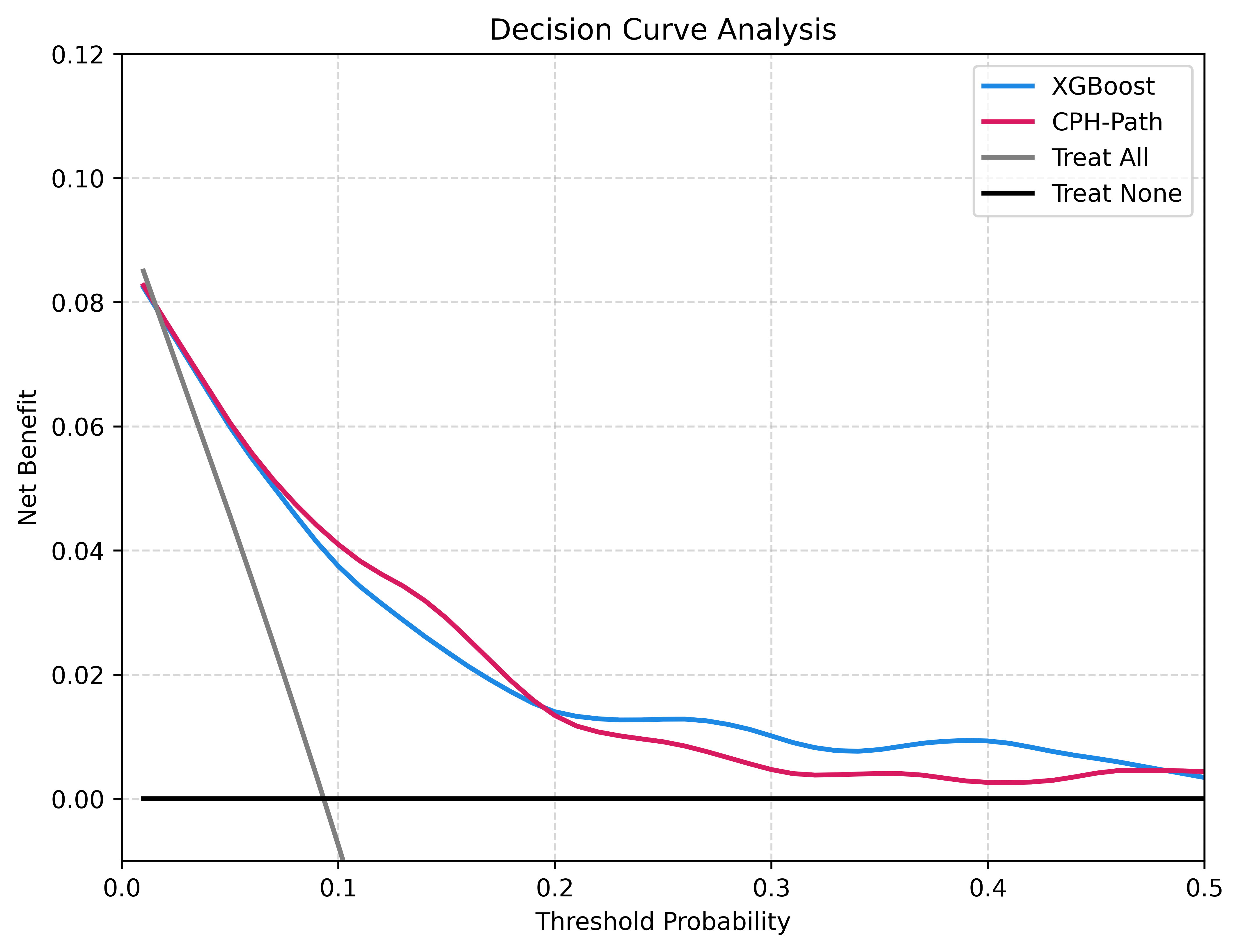

Supplement: Supplementary file 4 — Figure S2: Decision curve analysis curve showing the net benefit at different thresholds at three years for the XGBoost model compared to the CPH‐Path model against the treat all and treat non options. Abbreviations: CPH‐Path: pathology‐based Cox Proportional Hazards, XGBoost: Extreme Gradient Boosting. [file BCO2-7-e70234-s004.png]

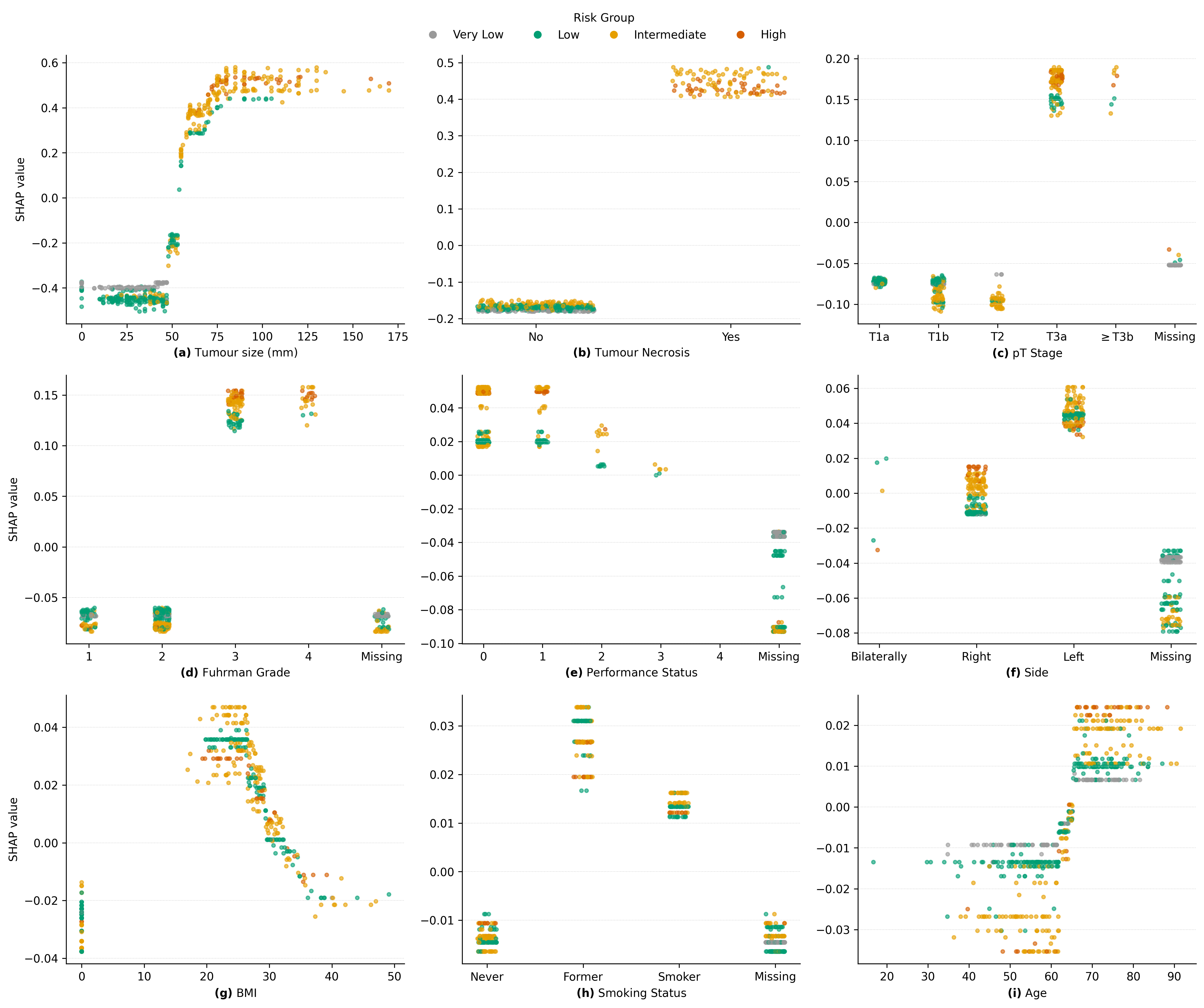

Supplement: Supplementary file 5 — Figure S3: SHAP dependence plots for the XGBoost model shown on the independent test set, with patients coloured by their risk group: Very Low (grey), Low (green), Intermediate (yellow), High (red). Abbreviations: XGBoost: Extreme Gradient Boosting, SHAP: Shapley Additive Explanations. [file BCO2-7-e70234-s001.png]
